# The Impact of Worry on Attention to Threat

**DOI:** 10.1371/journal.pone.0013411

**Published:** 2010-10-14

**Authors:** Desmond J. Oathes, Christian M. Squillante, William J. Ray, Jack B. Nitschke

**Affiliations:** 1 Department of Psychiatry and Behavioral Sciences, Stanford University, Stanford, California, United States of America; 2 Waisman Laboratory for Brain Imaging and Behavior, Departments of Psychiatry and Psychology, University of Wisconsin – Madison, Madison, Wisconsin, United States of America; 3 University of Pennsylvania Health System, Philadelphia, Pennsylvania, United States of America; 4 Department of Psychology, Pennsylvania State University, University Park, Pennsylvania, United States of America; Chiba University Center for Forensic Mental Health, Japan

## Abstract

Prior research has often linked anxiety to attentional vigilance for threat using the dot probe task, which presents probes in spatial locations that were or were not preceded by a putative threat stimulus. The present study investigated the impact of worry on threat vigilance by administering this task during a worry condition and during a mental arithmetic control condition to 56 undergraduate students scoring in the low normal range on a measure of chronic worry. The worry induction was associated with faster responses than arithmetic to probes in the attended location following threat words, indicating the combined influence of worry and threat in facilitating attention. Within the worry condition, responses to probes in the attended location were faster for trials containing threat words than for trials with only neutral words, whereas the converse pattern was observed for responses to probes in the unattended location. This connection between worry states and attentional capture by threat may be central to understanding the impact of hypervigilance on information processing in anxiety and its disorders.

## Introduction

Anxiety has been consistently linked theoretically to anticipation of potential aversive events or ‘threats’ [1]–[9]. A readiness to evaluate potential threats in the environment, commonly referred to as vigilance, suggests an attentional disposition associated with anxiety. A propensity to evaluate potential threats has been a useful model to explain physiological findings in anxiety including amygdala reactivity [10]–[12], connectivity of the amygdala with frontal and parietal cortices [13], [14], event related brain potentials [15], [16], and probes of the corticospinal motor system [17].

Behaviorally, vigilance has been most often defined by response times to presentations of threat-relevant stimuli. The dot probe task is commonly used to study vigilance in anxiety. Trials are comprised of a pair of words, one of which is followed by a neutral ‘dot’ probe. Threat trials include one threat word and one neutral word. On neutral trials, both words are neutral. The conventional analytic approach discards neutral trials and operationalizes threat vigilance by comparing response times for probes in the same location as the threat word to response times for probes in the same location as the neutral word [4], [18]–[23]. Many versions of the dot probe task have been used to study anxiety with variations including response type (choice or simple), stimulus type (pictures, words), stimulus pair locations (top/bottom, left/right, random), duration of stimulus presentations (subliminal to several seconds), and whether attention is unrestricted or directed to a particular stimulus location. A recent meta-analysis of dot probe task studies encompassing many of these paradigm variations found that both state and trait anxiety were associated with faster response times for probes in the same spatial location as previously presented threat stimuli than for probes in a different spatial location from the threat stimuli [19].

An alternative measure of threat vigilance compares the presence of a threat stimulus (trials with one threat word and one neutral word) to the absence of a threat stimulus (trials where both words are neutral), irrespective of spatial location [24]. The prediction here is that anxiety would be associated with faster response times for probes following displays that include threat stimuli than for probes following displays that include only neutral stimuli. In the present study, we tested the sensitivity of both measures of threat vigilance to a reliable experimental manipulation of worry, an important form of anxiety with content focused on concerns about unpleasant scenarios that might occur in the future.

Worry inductions increase reports of anxiety and unpleasant affect as well as decrease positive affect in clinically anxious patients and in non-psychiatric control participants [25]–[27]. Worry has also been associated with specific physiological profiles, as measured by electroencephalography [26], electromyography [17], positron emission tomography [28], and functional magnetic resonance imaging [29]. Worry states have been theoretically linked to a pervasive bias for threat detection [30]. Based on evidence that worry is a similar process for chronic worriers and for people reporting minimal worry [31], [32], it was hypothesized that a worry induction in non-worriers would facilitate responses to threat on the dot probe task and that these findings would carry relevance for conclusions drawn from the dot probe literature in GAD patients [4], [33] and other forms of anxiety [19]. Performance on the dot probe task has been manipulated in multiple ways [34]–[36], but this is the first study to directly manipulate worry or other forms of anxiety and test the effect on threat vigilance using this task.

In the present study, we induced worry states in participants reporting typical non-clinical levels of trait worry using a protocol that has been reliably used to induce worry in anxious and non-anxious individuals [17], [25]–[27]. For the conventional measure of threat vigilance using the dot probe task, it was predicted that worry would result in faster responses than mental arithmetic to probes in the same attended location as threat words. Mental arithmetic was selected as a control condition to contrast with the worry condition because both involve high cognitive load and at least mild emotional stress but only worry has been theoretically linked to vigilance for threat [18]. For the alternative measure of threat vigilance, we predicted that worry would result in faster responses to probes in the attended location than mental arithmetic on trials containing threat words, regardless of the location of the threat word, but not on trials containing only neutral words.

## Methods

### Ethics Statement

All participants signed informed consent documents approved by the institutional review board of the Pennsylvania State University. Research was conducted according to principles expressed in the Declaration of Helsinki and as approved by the institutional review board of the Pennsylvania State University.

### Participants

Fifty-six participants (half female) were undergraduate students at Penn State University in introductory psychology courses (ages 18–28, *M* = 18.75, *SD* = 1.54). All participants were administered the Penn State Worry Questionnaire (PSWQ) [37], a 16-item instrument that assesses trait levels of chronic uncontrollable worry. Participants were selected if their scores on the PSWQ were between 20 and 50 (*M* = 37.8, *SD* = 7.49). This range was chosen to represent worry from a non-anxious group that was not likely to be repressing anxiety or unusually apathetic to worry symptoms. Participants with a previous history of generalized anxiety disorder, panic disorder, or other mental illness as determined by self-report were excluded from this study. Following the conclusion of the experimental session, participants were given a debriefing form approved by the Psychology Department and institutional review board of the Pennsylvania State University.

### Materials

A personal computer (Pentium II Processor) running Eprime stimulus presentation software (Psychology Software Tools, Inc., Pittsburgh, PA) equipped with a keyboard for participant responses was used for the dot probe task. Threat trials were comprised of 96 threat words paired with emotionally neutral words matched for frequency of English usage and length. All words were obtained from MacLeod and McLaughlin [38]. Identical to the dot probe task employed by MacLeod et al. [4], the location of the threat word, its paired neutral word, and the subsequently presented probe were counterbalanced and randomly ordered so that each had equal probability of being in the top or bottom location on a given trial. Thus, two factors were independently varied on the threat trials: threat word location and dot probe location. The combination of these two factors resulted in four possible response conditions: threat on top with probe on top, threat on top with probe on bottom, threat on bottom with probe on bottom, and threat on bottom with probe on top. Neutral trials were identical in structure to threat trials except that they were comprised of an additional 96 word pairs featuring two neutral words. Filler trials were comprised of neutral word pairs similar to the neutral trials but were not followed by dot probes. None of the neutral words used for the filler trials were presented on threat or neutral trials. Presentations of threat, neutral, and filler trials were randomly ordered.

The words for the threat and neutral trials were divided into two equivalent lists so that no words were repeated within or across the worry and arithmetic conditions. All 96 neutral word pairs for the filler trials were presented for both conditions. Thus, each condition contained 192 trials of word pairs (48 threat, 48 neutral, and 96 filler trials). The 192 trials for each condition were presented in 24 blocks of 8 trials (6 blocks each for threat and neutral trials, and 12 blocks for filler trials). Each word pair was presented on the screen for 500 ms, with words presented in black capital letters, 18 mm high. On threat and neutral trials, an asterisk (the dot probe) appeared in place of one of the two words and remained on the screen until a response (pressing the space bar) had been made, at which point the next pair followed after a 1s delay. On filler trials, subsequent word pairs were presented after a 1s delay.

### Procedure

After signing the consent form, participants were asked to think of a worry topic of current concern to them that they could worry intensely about for several minutes total during the course of the experiment. Our and others' work have repeatedly established that this type of worry induction reliably facilitates future-oriented, anxious thoughts that are both unpleasant and engrossing for non-anxious individuals as well as for those meeting criteria for affective disorders [17], [25]–[27], [35]. All participants confirmed that they were able to generate worry topics of current concern. Participants were then seated at a fixed distance from the computer screen to ensure that the distance between words on the vertical axis was less than 2 degrees of visual angle. The following instructions were presented on the screen:

In this experiment you are going to see words presented on the screen in pairs. One word will appear just above the center of the screen, and one just below. Please read the top word of each pair aloud as soon as it appears. Sometimes when the two words disappear an asterisk (*) will remain either in the area where the top word appeared or in the area where the bottom word appeared. When you see this asterisk, press the space bar as quickly as possible.

Participants completed a short practice session featuring twelve trials (using words not employed in the main experiment). By the end of the practice session, all participants were able to appropriately respond when probes were presented and withhold responses when probes were not presented. The experimenter confirmed consistent attention by participants to the top word via monitoring of participants' verbal responses. During the practice session, the experimenter remained in the room to answer any questions about instructions presented on the screen and to monitor accuracy on practice trials.

Following the practice session, participants began one of two conditions, the order of which was counterbalanced across participants. In one experimental condition, participants were asked to worry, using the following instructions on the screen:

Now we would like you to worry about the topic that you chose earlier in the experiment. Please worry about this topic as intensely as you can, in the way that you usually worry, until you are asked to stop worrying. After a short period of worrying, the experimenter will resume the same experiment you have been working on thus far.

In the other condition, participants were asked to engage in mental arithmetic, using the following instructions on the screen:

For a short period, we would like you to do a mental subtraction in your head. At the end of this period, we will ask for the number you were last on. The number to start on is 1,320 and we would like you to subtract 7 from this number again and again in your head until we ask you to stop doing the subtraction. If you forget the number you were last on, think back to the last number you remember and continue subtracting.

Each set of instructions was presented on the screen for as long as the participant needed to read it (approximately 30 s). In between each block of 8 trials, participants were presented with instructions on the screen to either “continue worrying” or “continue subtracting.” These instructions remained on the screen for 30 s and were immediately followed by the next block of 8 trials. Our hypotheses concerned the effects of worry compared to mental arithmetic on the dot probe task, which was our behavioral measure of threat vigilance. Having the inductions and dot probe blocks contiguous in time without breaks for assessing affective self-report influences of the inductions was essential for maintaining the carry over effects of the inductions to the threat vigilance task. At the conclusion of the experimental runs, participants were given a debriefing form and any questions were answered.

### Data Analysis

Probe detection latencies of less than 100 ms (premature responses) and greater than 3000 ms (delayed or missed responses) occurred on 1% of the trials and were excluded from analyses. Mean response times for each condition and trial type were calculated for each participant separately. To test the alternative operationalization of threat vigilance comparing the presence of a threat stimulus (trials with one threat word and one neutral word) to the absence of a threat stimulus (trials with two neutral words), threat-related facilitation of reaction times was assessed by an omnibus Condition (worry, arithmetic) × Probe Location (top, bottom) × Trial Type (threat, neutral only) repeated-measures ANOVA and appropriate posthoc analyses. Note that Threat Word Location (top, bottom) cannot be included as a factor in this omnibus analysis because neutral only trials for the Trial Type factor do not include threat words. The conventional operationalization of threat vigilance used in prior research using the dot probe task was tested by a Condition (worry, arithmetic) × Probe Location (top, bottom) × Threat Word Location (top, bottom) repeated-measures ANOVA on the threat trials. Pearson's product moment correlations assessed associations among performance metrics and PSWQ scores. Two-tailed tests were used throughout. Cohen's *d* statistic for effect sizes was computed for individual contrasts [39].

## Results

**Figure 1:**
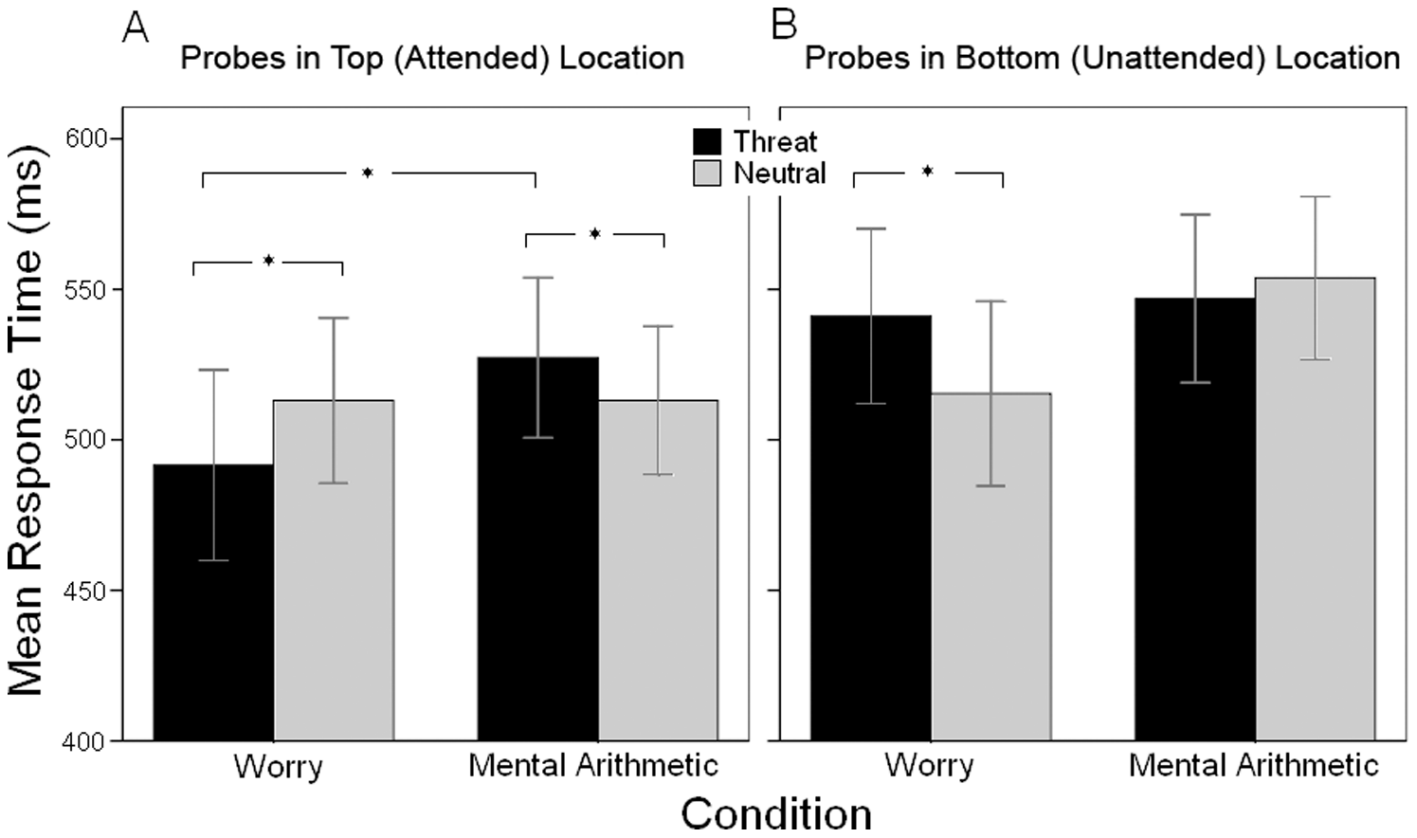
Response times to probes. Response times to probes following word pair presentations that either included a threat word (Threat) or did not include a threat word (Neutral). Graphs illustrate response times to probes in the attended (A) and unattended (B) locations following worry inductions or following mental arithmetic. Bars represent 95% confidence intervals [73] around the means after adjusting for between-subject variance [74]. *p<0.05.

For the omnibus ANOVA, significant main effects were found for Condition, *F*(1,55) = 7.75, *p*<0.01, and for Probe Location, *F*(1,55) = 53.80, *p*<0.001. Worry was associated with faster response times compared to mental arithmetic, and probes presented in the top (attended) location were responded to faster than those in the bottom location (Table 1). Central to study hypotheses, there was a Condition × Probe Location × Trial Type interaction, *F*(1,55) = 27.72, *p*<0.001 (Figure 1). No other main effects or interactions were significant for the omnibus ANOVA.

**Table 1:** Response times to probes following either worry induction or mental arithmetic.

	Threat Word Location on Threat Trials		
Probe Location	Top	Bottom	Neutral Trials
	*Worry*		
Top	494.74 (117.67)	488.44 (125.33)	513.07 (96.86)
Bottom	529.70 (108.26)	548.36 (123.75)	515.36 (109.23)
	*Mental Arithmetic*		
Top	521.70 (106.04)	532.93 (96.21)	513.11 (91.89)
Bottom	533.86 (98.21)	559.99 (112.33)	553.77 (100.98)

Mean response times and standard deviations (in parentheses) to probes are shown as a function of trial type (threat, neutral), threat word location (top, bottom), probe location (top, bottom), and condition (worry, mental arithmetic). ‘Threat trials’ include one threat word and one neutral word; ‘neutral trials’ include two neutral words and no threat word.

For the Condition × Probe Location × Threat Word Location ANOVA on threat trials, main effects were observed for Condition, *F*(1,55) = 7.92, *p*<0.01, and for Probe Location, *F*(1,55) = 43.82, *p*<0.001. As for the omnibus ANOVA above, worry was associated with faster response times than arithmetic, and probes presented in the top location were responded to faster than probes in the bottom location. There was also a main effect of Threat Word Location, indicating that threat words presented in the top (attended) location were followed by faster responses to subsequent probes than threat words presented in the bottom (unattended) location, regardless of probe location, *F*(1,55) = 7.64, *p*<0.01. A Condition×Probe Location interaction, *F*(1,55) = 14.24, *p*<0.001, for this ANOVA on threat trials qualified the 3-way interaction described above for the omnibus ANOVA. As hypothesized for the alternative operationalization of threat vigilance, worry was associated with faster response times than arithmetic to probes in the attended location, regardless of whether the threat word was presented in the attended or unattended location, *t*(55) = 3.54, *p*<0.001, *d* = 0.77 (Figure 1). For probes in the unattended location, there was no significant difference between the conditions, *t*(55) = 0.94, *p* = 0.35, *d* = 0.12. The response times to probes in the attended location for worry were also faster than to probes in the unattended location for either worry, *t*(55) = 6.50, *p*<0.001, *d* = 0.44, or arithmetic, *t*(55) = 5.45, *p*<0.001, *d* = 0.50. No other effects were significant, including the Condition×Threat Word Location×Probe Location interaction, *F*(1,55) = 3.14, *p* = 0.25, which serves as the test of the conventional operationalization of threat vigilance positing faster responses to probes in the same attended location as threat words [4], [33]. The absence of this interaction also indicated that worry was not associated with difficulty disengaging attention from threat.

The Condition × Probe Location ANOVA on neutral trials revealed the same Condition and Probe Location main effects indicating that worry was associated with quicker response times than arithmetic, *F*(1,55) = 6.11, *p*<0.05, and that probes presented in the top location were responded to faster than probes in the bottom location, *F*(1,55) = 19.57, *p*<0.001. The critical Condition×Probe Location interaction was also significant, *F*(1,55) = 19.77, *p*<0.001. As hypothesized for the alternative operationalization of threat vigilance, the faster response times for worry than arithmetic reported above for threat trials were not present for neutral trials, *t*(55) = 0.00, *p* = 0.997, *d* = 0.00 (Figure 1). Instead, the response times to probes in the unattended location for arithmetic were slower than for worry, *t*(55) = 4.11, *p*<0.001, *d* = 0.36, and were slower than to probes in the attended location for either worry, *t*(55) = 4.63, *p*<0.001, *d* = 0.40, or arithmetic, *t*(55) = 5.31, *p*<0.001, *d* = 0.42. No other effects were observed for trials with only neutral words.

Study hypotheses were also supported by ancillary pairwise comparisons for key contrasts. The worry condition was associated with faster responses to probes in the attended location for threat than neutral trials, *t*(55) = 3.10, *p*<0.005, *d* = 0.19 (Figure 1A). The opposite was observed for the unattended location, with the worry condition accompanied by slower responses for threat than neutral trials, *t*(55) = 3.92, *p*<0.001, *d* = 0.23 (Figure 1B). In contrast, mental arithmetic was accompanied by slower response times to probes in the attended location for threat than neutral trials, *t*(55) = −2.34, *p*<0.05, *d* = 0.15 (Figure 1A), and there was no difference for probes in the unattended location, *t*(55) = 1.20, *p* = 0.24, *d* = 0.07 (Figure 1B).

As done in prior studies using the dot probe task [4], [20], [33], [40]–[41], an attention bias index was computed as the difference between responses to attended and unattended threat word locations (average difference between matched and non-matched threat/probe locations). The worry and arithmetic conditions were not significantly different for this bias index, *t*(55) = 0.56, *p* = 0.58. In addition, the PSWQ was not correlated with this bias index (Worry condition: *r* = 0.11, *p* = 0.40; Math condition: *r* = 0.03, *p* = 0.81; overall: *r* = 0.11, *p* = 0.44) or other related metrics of behavioral performance (e.g. Worry condition for matched threat and probe locations: *r* = 0.09, *p* = 0.50; Worry condition for threat trials: *r* = 0.08, *p* = 0.58; Math-Worry for threat trials: *r* = 0.08, *p* = 0.56). There were also no gender differences for the PSWQ or the above behavioral measures (all *p*s>0.27).

## Discussion

The dot probe is widely regarded as a definitive behavioral task for detecting threat vigilance associated with anxiety. The traditional analysis strategy for this task compares response times when threat stimuli and probes are in the same spatial location to response times when the threat and probe are in different spatial locations. Our contention is that the comparison of trials containing threat stimuli to trials that do not include threat stimuli is a relatively untapped resource [24], [41] for understanding how the mere presence of threat may influence behavior. This expanded analysis strategy is consistent with theoretical models of anxiety which have emphasized the link between anxiety and attunement to potential threats in the environment. In the present study, we found that comparing trials with threat words to trials with only neutral words was sensitive to the detection of a threat bias for the anxiety condition (worry induction), whereas the conventional analysis strategy analyzing matches in threat and probe locations for trials with threat words was not. The presence of a threat word, whether in the attended or unattended location, was followed by faster responses to a probe in the attended location during the worry condition than during the mental arithmetic control condition. Moreover, within the worry condition, responses were faster to probes in the attended location than to probes in the unattended location, whereas no differences were observed for the mental arithmetic condition. These results suggest that the act of worrying is sufficient to facilitate an attentional bias to threat and enhances the beneficial effects of attention to a particular spatial location.

Initial findings by MacLeod et al. [4] suggested that the location of the threat word in the attended location is critical, whereas findings here indicate that the mere presence of threat in either the attended or unattended location affects subsequent behavioral responses. Comparisons of threat and neutral trials are not typically reported in dot probe studies, but such comparisons bolster claims regarding the impact of threat on behavior [24]. Theories of anxiety [1]–[5], [7]–[9], [32] and worry [25], [42] suggest that vigilance is a key factor in anxiety pathology, consistent with evidence across a range of physiological indicators [12], [17], [26], [29], [43]–[44]. Alternatively, others have argued that a difficulty to disengage attention from threat is the aspect of attention [45] that is likely to be biased in anxiety and worry [24], [46]–[48]. The facilitated responses for the combination of worry, attention, and threat shown in Figure 1 implicated vigilance rather than disengagement aspects of attention [45]. Specifically, vigilance was indicated by worry being accompanied by faster responses to probes in the attended location following threat words in either spatial location. Disengagement, on the other hand, would be indicated by worry being accompanied by slower responses to probes in a different location than the threat word, which was not found. The dot probe task may be particularly sensitive to the impact of a recent worry experience on vigilance, extending prior research showing the influence of worry on subsequent processing in other domains [32], [49]–[51]. Assuming that anxious individuals and especially GAD patients may regularly experience worry, our dot probe findings indicate that these frequent experiences of worry may directly contribute to preferential vigilance for threat cues in the environment for these individuals.

Complementing speeded responses to the attended location following threat words for the worry condition, we also found that worry was accompanied by slower responses to probes in the unattended location when following a threat word than when only neutral words were presented. This slowing is consistent with prior research documenting interference from worry on subsequent processing of unrelated material [27], [49]. However, given that the threat word location did not qualify this slowing, findings here do not support specific disengagement failures associated with anxiety, as noted above. A different pattern was observed for mental arithmetic, with slower responses to probes in the attended location when following a threat word than when only neutral words were presented (and no response differences for the unattended location). This perhaps reflects a lack of congruence between mental arithmetic and threat processing, as opposed to the congruence between worry and threat processing. In support of this interpretation, lack of congruence in emotional content is associated with slower response times and poorer accuracy than processing congruent information [52]. Alternatively, the degree of carry over influence from the worry induction could have been higher than from the mental arithmetic manipulation, potentially resulting in effects diminishing more rapidly for the arithmetic condition than for the worry condition.

GAD patients typically have slower response times overall compared to individuals with no psychiatric diagnosis [4], [33], whereas worry here was associated with faster response times than mental arithmetic collapsing across threat and neutral trials. Slowed responses in GAD patients may be related to general problems with sustained attention in clinical populations or with the interference of comorbid depression on dot probe performance [53], [ see also 54]. In a dot probe experiment, McKay [55] found that chronic worriers not drawn from a clinical population made faster responses overall than non-worriers. In addition, across both groups, a pattern of faster response times for threat than neutral trials was observed following worry induction, as confirmed here, but speeded responses did not follow a positive mood induction. Accordingly, we predict that our findings will most likely extend to populations who have high levels of chronic worry but who present with few confounding additional factors (e.g., medication, co-morbid depression).

Along these lines, the present results are not likely due to the influence of depression or other forms of negative affect that co-occur with worry [4], [22], [33], [56]–[60]. Depression typically has not been associated with an attentional bias for threat [4], [56], [61]–[62]. Self-report measures of state and trait anxiety that psychometric studies have demonstrated are primarily indicators of negative affect [63], [64] are uncorrelated with dot probe performance [18], [33], [34]. The PSWQ in the present study was also not correlated with measures of threat vigilance, indicating that behavior may yield evidence of threat vigilance not effectively assessed by common self-report measures. The specificity of worry inductions for promoting threat vigilance should be assessed in future studies by comparisons to other inductions (e.g., fear, anger, sadness) and measures (e.g., cognitive flexibility, emotion regulation). We did not assess self-report influences at the conclusion of induction/dot probe blocks given the unreliability of retrospective rather than experiential reports of affect [65]. However, the average response time differences across tasks provided evidence of vigilance according to our manipulations, and future research may uncover an appropriate instrument to tap the experiential correlates of these effects.

Interestingly, the causal flow of worry leading to increases in threat vigilance may be reversible. Evidence suggests that training high worriers to attend to nonthreatening words reduces negative thought intrusions during worry [66]. Similarly, focusing on benign meanings of potentially aversive information reduces negative thought intrusions and anxiety in high worriers [67], [68]. Manipulating dot probe stimulus probe contingencies so that probes replace neutral words on 90–100% of trials has been shown to reduce threat biases and decreased anxiety symptoms in chronic worriers [69] and GAD patients [70]. A valuable future study bridging the present study with these recent findings for attention training and modification could test whether similar strategies mitigate the effects found here for worry induction.

The specificity of the behavioral findings for the worry induction provides evidence for how worry affects behavior. However, additional measures could enrich our understanding of the mechanisms and effects of the worry inductions. For example, assessing worry induction success and the degree of distress caused by the worry might inform how worry influences subsequent attention. Similarly, physiological data such as autonomic reactivity would provide valuable complementary understanding of the worry process and subsequent behavioral effects. Future research is also needed to determine whether findings for worry here extend to other versions of the dot probe task, such as those using pictures instead of words, different stimulus locations and durations, stimulus discrimination rather that stimulus localization, and unrestricted attention rather than instructions to attend to a particular location. In particular, directing attention to the top location in the present study might have interfered with the previously reported effects comparing matched and non-matched threat/probe spatial locations.

Our study design and findings bypass three criticisms that have been leveled at previous dot probe studies. One criticism is that although the verbal modality may be ideally suited to study anxiety [25], [58], anxious individuals may be more facile with threat words than non-anxious individuals, yielding a potential confound in using these words to study group differences for threat vigilance [71]. In this study, only non-anxious participants were included, and anxiety was manipulated on a within-subjects basis. Second, reliability concerns pertaining to the use of the dot probe task in non-clinical populations were obviated by experimental manipulations that affect attentional vigilance in a state-dependent manner, as shown here and elsewhere [36], [72]. Third, the traditional dot probe task used in this study does not guarantee that attention will stay on the presented words in the attended location for the full 500-ms durations [46], [58]. Findings here do not depend on attention remaining fixed on the presented words in the attended location, given that the presence of a threat word in either location influenced response times. Future research could add to the present findings by using eyetracking measures to explore the influence of worry on maintaining overt visual attention to threat stimuli while they are presented.

The present study extends previous research using the dot probe task to investigate threat vigilance associated with anxiety by highlighting the specific contribution of worry states to vigilance for threat. Comparisons between worry and a mental arithmetic control condition as well as comparisons between trials with threat words and trials with only neutral words point to the influence of the combination of worry and threat on behavior. Present findings suggest that the confluence of worry and threat is instrumental in creating an attentional bias to threat similar to findings from studies of state and trait anxiety including GAD. Frequent, long-lasting worry states in clinical populations may contribute to patterns of hypervigilance in these individuals. Psychotherapy techniques that focus on reducing worries would thus be expected to mitigate the vigilance and preferential attention to threat that characterize GAD and other forms of anxiety.
